# A discordant of blood glucose analysed by Glucometer and the Central lab method in an infant with Galactosemia

**DOI:** 10.1186/1687-9856-2013-S1-P178

**Published:** 2013-10-03

**Authors:** Sukumarn Siripunthana, Taninee Sahakitrungruang, Suttipong Washarasindhu, Kanya Suphapeetiporn, Vichit Supornsilchai

**Affiliations:** 1 Division of Pediatric Endocrinology, Department of Pediatrics, Faculty of Medicine, Chulalongkorn University, Bangkok 10330 Thailand; 2 Division of Medical Genetics and Metabolism, Department of Pediatrics, Faculty of Medicine, Chulalongkorn University, Bangkok 10330, Thailand

## 

Galactosemia is an autosomal recessive disorder, which caused by a deficiency of one of three enzymes that involved in the metabolism of galactose; galactokinase (GALK), galactose-1-phosphate uridyltransferase (GALT) and uridine-diphosphate galactose-4'epimerase (GALE). Clinical manifestations including poor feeding, jaundice, hepatomegaly, vomiting, hypoglycemia, convulsions, failure to thrive, cataracts, bleeding disorder, renal failure, liver cirrhosis, mental retardation and increased risk for *E. coli* sepsis,develop after milk feeding. We reported an infant who presented with prolonged hyperbilirubinemia and different result of blood glucose between glucometer and the central lab which is an unusual presentation of galactosemia.

A 11- day-old male infant born to non-consanguineous parents presented with jaundice, poor feeding, poor weight gain, vomiting and drowsiness when he was 6-day-old. On physical examination revealed lethargic, icteric sclerae and hepatomegaly. Cataract examined by the opththalomologist was not found. Routine work up for the causes of neonatal jaundice was found to be normal. LFTs showed TB of 26.8, DB 3.57 mg/dL, SGOT 82, SGPT 50 and ALP 871U/L. TFTs showed FT_4_ of 1.62 ng/dL, TSH 4.91 mu/L. Electrolytes and coagulogram were normal. His blood sugar was 220 mg/dL by Glucometer (Accu-chek Performa) without any symptoms and signs of hyperglycemia and glucosuria, but it was 60 mg/dL by the central lab method. Galactosemia was confirmed by the high level of urine Galactitol and the genetic analysis of Galactose-1-phosphate uridyl transferase (GALT) found compound heterozygous mutation of p.R259W in exon 8 and p.E340K in exon 10 which is a known mutation. One day after soy-based formula was started, the bilirubin levels were gradually reduced to normal for 1 week and the discrepancy of blood glucose between Glucometer and the central lab method was resolved. Sepsis can not be totally excluded by this clinical setting, antibiotic was started to cover the organism which is common for this age group including *E Coli*.

This paper reported infant with Galactosemia who presented with an unusual finding of the discordance between blood sugar levels analysed by Glucometer and the central lab method due to some Glucometer analyse other form of sugar especially Galactose and its metabolites.

